# Supplementary material for: Causal Relationship between Aging and Anorexia Nervosa: A White-Matter-Microstructure-Mediated Mendelian Randomization Analysis
Source: Biomedicines. 2024 Aug 16;12(8):1874. doi: 10.3390/biomedicines12081874 (PMC11351342; doi:10.3390/biomedicines12081874)
Supplement: Supplementary file 1 [file biomedicines-12-01874-s001.zip › Supplementary Figure/Figure S4/Singe SNP plot of MR analysis for TL to ubm-b-2068.pdf]

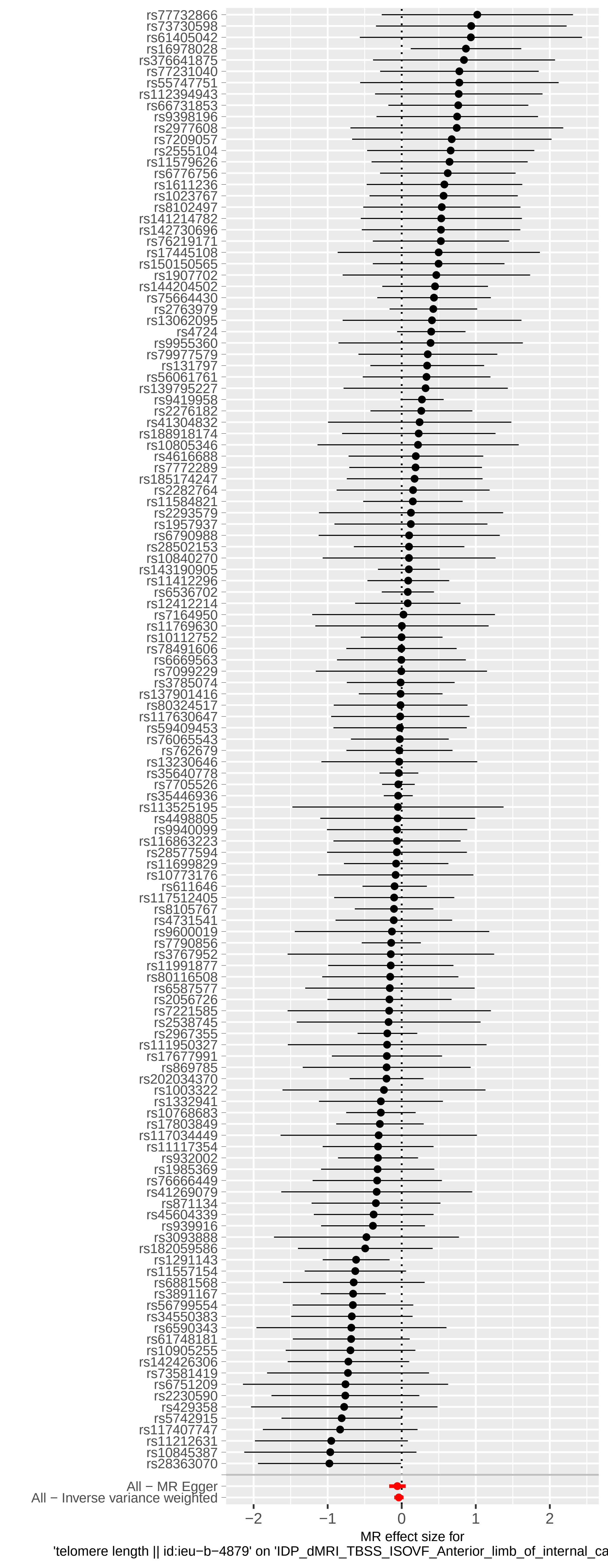

rs77732866
rs73730598
rs61405042
rs16978028
rs376641875
rs77231040
rs55747751
rs112394943
rs66731853
rs9398196
rs2977608
rs7209057
rs2555104
rs11579626
rs6776756
rs1611236
rs1023767
rs8102497
rs141214782
rs142730696
rs76219171
rs17445108
rs150150565
rs1907702
rs144204502
rs75664430
rs2763979
rs13062095
rs4724
rs9955360
rs79977579
rs131797
rs56061761
rs139795227
rs9419958
rs2276182
rs41304832
rs188918174
rs10805346
rs4616688
rs7772289
rs185174247
rs2282764
rs11584821
rs2293579
rs1957937
rs6790988
rs28502153
rs10840270
rs143190905
rs11412296
rs6536702
rs12412214
rs7164950
rs11769630
rs10112752
rs78491606
rs6669563
rs7099229
rs3785074
rs137901416
rs80324517
rs117630647
rs59409453
rs76065543
rs762679
rs13230646
rs35640778
rs7705526
rs35446936
rs113525195
rs4498805
rs9940099
rs116863223
rs28577594
rs11699829
rs10773176
rs611646
rs117512405
rs8105767
rs4731541
rs9600019
rs7790856
rs3767952
rs11991877
rs80116508
rs6587577
rs2056726
rs7221585
rs2538745
rs2967355
rs111950327
rs17677991
rs869785
rs202034370
rs1003322
rs1332941
rs10768683
rs17803849
rs117034449
rs11117354
rs932002
rs1985369
rs76666449
rs41269079
rs871134
rs45604339
rs939916
rs3093888
rs182059586
rs1291143
rs11557154
rs6881568
rs3891167
rs56799554
rs34550383
rs6590343
rs61748181
rs10905255
rs142426306
rs73581419
rs6751209
rs2230590
rs429358
rs5742915
rs117407747
rs11212631
rs10845387
rs28363070
All − MR Egger
All − Inverse variance weighted
−2
−1
0
1
2
MR effect size for
'telomere length || id:ieu−b−4879' on 'IDP_dMRI_TBSS_ISOVF_Anterior_limb_of_internal_ca
